# Edible *Pleurotus eryngii* Papery Food Prepared by Papermaking Process

**DOI:** 10.3390/foods11213514

**Published:** 2022-11-04

**Authors:** Shuang Lv, Xiaolin Zhu, Zhenbin Liu, Liangbin Hu, Dan Xu, Bimal Chitrakar, Haizhen Mo, Hongbo Li

**Affiliations:** 1 School of Food Science and Engineering, Shaanxi University of Science and Technology, Xi’an 710021, China; 2 College of Food Science and Technology, Hebei Agricultural University, Baoding 071001, China

**Keywords:** mycelium, papery food, papermaking process, *Pleurotus eryngii*

## Abstract

The objective of the current study was to evaluate the feasibility of papery food with *Pleurotus eryngii* (*P. eryngii*) as a raw material using the papermaking process. The physical, chemical, structural, and thermal degradation properties were studied as well as the sensory evaluation of the papery food from *P. eryngii* mycelia (PMP), stems (PSP), caps (PCP), and whole fruiting bodies (PEP). The results indicated that the colors from PSP, PCP, and PEP were clearly different from PMP. Thicker PSP and PMP had a smoother surface and better crispness compared to PCP. Moreover, PSP had better moisture resistance and thermal decomposition performance compared to the other groups. Nutritional composition and Fourier-transform infrared spectroscopy suggested abundant polysaccharide and protein content in all of the papery food. Finally, sensory evaluation showed that the formability, mouth feel, and overall palatability of PSP and PMP were more popular among consumers. Overall, this study provides a novel method for the preparation of papery food and provides a potential new mechanism for the further development and utilization of the fruiting bodies and mycelium of *P. eryngii*.

## 1. Introduction

Papery food, also known as edible special paper, has several desirable properties such as longer shelf life, good taste, and nutrition. Usually, it is consumed as a side dish and snack food. The products not only retain the flavor and nutritional qualities of raw materials, but also facilitate transportation and storage. At present, studies on papery food with fruits and vegetables as raw materials are being actively performed worldwide [1,2]. Saxena et al. [2] prepared jackfruit bulb crisps with good texture and color by a combination of freeze drying (FD) and hot air drying (HAD), which has the potential for commercialization. However, using the fruiting bodies and mycelium of edible mushrooms as starting materials is scarcely reported.

*Pleurotus eryngii* is referred to as the king trumpet or king oyster mushroom and belongs to the *Pleurotus* genus [3]. It is native to the high mountains, grasslands, and deserts in southern Europe, North Africa, and central Asia. Nowadays, it is cultivated around the world. *P. eryngii* is known to be among the most commercially valuable species in the *Pleurotus* genus. It is widely consumed worldwide, not only due to its distinctive taste, texture, and longer shelf life, but also because of its nutritional and medicinal properties. The *P. eryngii* mycelium was reported to be used for the treatment of both humans and animals due to its antibacterial, antifungal, and antiprotozoal effects [4]. Considering that the wide range of sources and high edible and medicinal value of *P. eryngii*, the development of healthy food may be greatly promoted once the industrial scale of research and development of *P. eryngii* papery food has been formed.

Multiple studies over decades have demonstrated that *P. eryngii* has several biological functions, such as anti-tumor, anti-oxidation, hypolipidemic, and hypoglycemic activities, which are mostly associated with the presence of various active ingredients such as polysaccharides, proteins, peptides, dietary fibers, and vitamins [3,5,6]. Nevertheless, the fruiting body of *P. eryngii* requires a large amount of labor costs. Moreover, it demands a relatively longer growth period, which is highly susceptible to environmental conditions. Thus, increased attention has been paid to the mycelia of *P. eryngii* in recent years [5].

As the vegetative organ of the edible mushroom, mycelium is rich in proteins, carbohydrates, inorganic substances, dietary fibers, and other nutrients, whereas it is low in calories and fat content [7]. Liquid fermentation with a lower probability of pollution is a rapid and effective method to generate mycelia and metabolites by utilizing industrial and agricultural wastes [8]. The reason for using mycelia as an alternative factory source is because of their rapid growth, production of high amounts of biomass, and the widespread distribution of hyphae in the environment [4].

Most of the papery food items currently available in the market are prepared by film former coating, drying, demolding, and other processes, which are complex and time consuming [9,10]. Wang and Ma [1] prepared a novel edible paper from the insoluble dietary fiber of Chinese cabbage using the papermaking process and found that it has great potential in food and pharmaceutical packaging in the future. However, the edibility of products may be affected by the use of chemical reagents such as alkali solution and acetic acid in the preparation process. Inspired by the ancient Chinese papermaking technology that is divided into four major steps: raw material separation, pulping, papermaking, and drying [11], the current study made several improvements to the method of papery food preparation. The papery food produced by combining the processing technology with the papermaking process has a flatter appearance and a uniform water distribution compared to those produced using the existing method.

In the present study, a leisure papery food with certain healthcare functions was prepared from the mycelium (PM), stems (PS), caps (PC), and whole fruiting bodies (PE) of *P. eryngii* through a low-cost papermaking process. Moreover, the nutrient compositions, color, crispness, structural properties, moisture absorption rate, thermal degradation properties, and sensory evaluation of the papery food were measured to evaluate its feasibility. This study not only enriches the types of papery food available, but also expands the consumption market of *P. eryngii*, as well as providing a novel insight for the full utilization of the fruiting bodies and mycelium of edible mushrooms.

## 2. Materials and Methods

### 2.1. Materials

The strains of *P. eryngii* used in this study were a generous gift from Dr. Changtian Li (Jilin Agricultural University, Changchun, China), which were preserved on potato dextrose agar (PDA) medium at 4 °C and subcultured regularly to maintain their vitality. The cultures were incubated on PDA at 25 °C for 7 d. The growing mycelia were inoculated in 100 mL of the potato dextrose broth (PDB) medium and cultured in a constant-temperature incubator shaker (ZQPZ-85, Tianjin Leibo Terry Equipment Co., Ltd., Tianjin, China) at 25 °C and shaken at 150 rpm for 10 d. The resulting mycelia were then collected by vacuum filtration and washed three times with sterile distilled water.

Fresh *P. eryngii* were bought from a local supermarket in Xi’an, China. After cleaning, the mushroom stems were separated from the caps. The mycelia, stems, caps, and whole fruiting bodies were used as raw materials for preparing papery foods and part of them were saved for backup after vacuum freeze-drying (ALpHA2-4 LD plus, Marin Christ, Osterode, Germany).

All chemicals and reagents used in this research were of analytical grade and were purchased from Macklin (Shanghai Macklin Biochemical Co., Ltd., Shanghai, China), unless otherwise indicated.

### 2.2. Sample Preparation

Four grams of PM, PS, PC, and PE dry weight were mixed with 1 L distilled water, respectively, which was dispersed into fibrous form by a fluffer (IMT-SJ01, Dongguan Hengke Automative Equipment Co., Ltd., Dongguan, China). Then, the mixture was further dispersed with a beater (IMT-VL01, International Material Tester Co., Ltd., Dongguan, China) and then vacuum dried at 110 °C for 5 min after forming the sheets using a paper sheet former (AT-CZ-4, Shandong Annimet Instrument Co., Ltd., Jinan, China). The original water volume in the barrel of the paper sheet former was determined to be 0.6 L, and the bubbling number was three times. Finally, the sheets were carefully removed from the filter cloth to obtain the papery food of *P. eryngii* from the mycelia, stems, caps, and whole fruiting bodies.

### 2.3. Characterization

#### 2.3.1. Nutrient Composition Analysis

To determine the moisture content, the samples were oven dried at 105 ± 2 °C until a constant weight (DHG-9245AE, JIECHENG Experimental Instrument Co., Ltd., Shanghai, China) [12]. The total nitrogen content was determined by the Kjeldahl method (K1160, Hanon Advanced Technology Group Co., Ltd., Jinan, China) and the protein content was estimated by nitrogen values using a conversion factor of 6.25 [13].

Crude fiber was assayed according to the method of Lu et al. [14]. Briefly, papery food powder was successively digested by boiling 1.25% H_2_SO_4_ and 1.25% NaOH, filtered and washed with distilled water. The residue was oven dried to a constant weight and transferred to a muffle furnace (SX-17, ALCERA (SUZHOU) Co., Ltd., Suzhou, China) for incineration at 550 °C for 5 h. The formula for the crude fiber calculation was as follows:

$$Crude\;fiber\;content\;\left(\%\right)=\frac{\left(SMD-SMI\;\right)\times 100}{Sample\;Mass}$$

where:

*SMD* = sample mass after drying,

*SMI* = sample mass after incineration.

The total sugar content was determined by the Phenol-H_2_SO_4_ method according to the previous report of Ying et al. [15] with slight modifications. Briefly, the samples were mixed with 65 mL water-concentrated hydrochloric acid solution (10:3 *v*/*v*) and placed into a 100 °C water bath (OLB-WB26, Jinan OLABO Technology Co., Ltd., Jinan, China) for 3 h. The cooled solution was filtered and the volume was increased to 250 mL with distilled water. The reaction mixture consisted of 0.2 mL sample solution, 0.8 mL of distilled water, 1 mL of 5% phenol solution, and 5 mL of concentrated sulfuric acid, and was placed into a 30 °C water bath for 20 min. The absorbance was measured at 490 nm with a spectrophotometer (HIRP V1700G, HIRP International Trade Co., Ltd., Shanghai, China). D-glucose solutions were used for the calibration curve.

Total ash determination was performed on the dried samples. Weighed samples were pre-incinerated to smokeless at 525 °C in crucibles in a high-temperature furnace (HP-1000 W, JOAN LAB Equipment Co., Ltd., Ningbo, China) and then transferred to a muffle furnace. The samples were heated at 550 °C for several hours until they completely turned into ash and were free from black carbon particles. Finally, the crucibles were transferred into desiccators and cooled so that the samples could be weighed [12].

#### 2.3.2. Crispness Analysis

The 30 × 30 mm portions of the papery food were penetrated with a 0.64 cm diameter cylindrical stainless-steel probe (Type P/0.25S) by a texture analyzer (TA.XT plus, Stable Micro Systems, Godalming, UK). The pre-test and post-test probe running speed was set to 1.0 mm/s, while the test speed was set to 0.5 mm/s. The probe distance, trigger force and data acquisition rate were set as 10 mm, 5 g and 200 pps, respectively [16,17,18]. Three replicates were conducted for each sample. The force–displacement curves were recorded, and the numbers of positive peaks were obtained to calculate the number of spatial ruptures (*N_sr_*) of the papery food according to the following formula:

(1) $$N_{sr}=\frac{N_{0}}{d}$$

where *N*_0_ is the total number of positive peaks and *d* is the rupture distance (mm).

#### 2.3.3. Thickness, Density, and Color Analysis

After oven-drying at 60 °C for 48 h, the sheet samples were weighed with an analytical scale and average values were calculated. The sheets’ diameters were measured using a vernier caliper (530-101 N15, Mitutoyo, Tokyo, Minato City, Japan), while the thicknesses were obtained using a dial thickness gauge (GT-313-A1, GOTECH Testing Machines Inc., Taiwan, China). The sheet density was derived by dividing the mean sheet mass by the volume [19].

The color parameters, including a* (redness/greenness), b* (yellowness/blueness), and L* (lightness/darkness), were measured using a spectrocolorimeter (CM-5, Konica Minolta (China) Investment Ltd., Shanghai, China). Each piece of papery food was measured at least three times at different sites [20].

#### 2.3.4. Moisture Absorption Rate Analysis

The completely dried papery food was put into a dry flat glass weighing bottle (70 × 35 mm) and placed in an incubator (RK-TH-100, RIUKAI Instrument Technology Co., Ltd., Dongguan, China) with a constant temperature of 25 °C and 85% humidity. The weight of each papery food was obtained every 4 h. The moisture absorption rate was calculated according to the formula described in Kotturi et al. [21], as follows:

$$Moisture\;absorption\;rate=\frac{Final\;weight-Initial\;weight}{Initial\;weight\;}\times 100$$

#### 2.3.5. Thermogravimetric Analysis (TGA)

The thermal degradation properties of the papery food were assessed using a thermal gravimetric analyzer (Discovery TGA 55, TA Instruments, Newcastle, USA, DE) under a nitrogen atmosphere (nitrogen flow rate of 30 mL/min). About 4 mg of papery food samples were set in an alumina crucible with the temperature varying from 40 to 600 °C and a heating rate of 10 °C/min [22].

#### 2.3.6. Sensory Evaluation

The organoleptic properties of papery food were evaluated by a panel of twelve graduate students majoring in food science and engineering, with each sample assigned with their respective code. The evaluators were requested to assess the product for formability, color, mouth feel, and overall palatability using a 9-point hedonic scale ranging from 1 to 9, where 1 corresponded to “extremely dislike” and 9 corresponded to “extremely like” [23]. Formability can be defined as the integrity and smoothness of the papery food surface, the existence of cracks, and the uniformity of distribution. Mouth feel was defined as whether the papery food is crispy, has a pure taste, and has the unique flavor of *P. eryngii*.

#### 2.3.7. Fourier-Transform Infrared (FT-IR) Spectroscopy Analysis

FT-IR spectra were recorded using a Bruker Fourier-transform infrared instrument (VECTOR 22, Bruker (Beijing) Scientific Technology Co. Ltd., Beijing, China) with a deuterated triglycine sulfate detector and KBr beam splitter. The samples were ground in a mortar with liquid nitrogen and mixed separately with dried KBr at a ratio of 1:50. The powder was thoroughly mixed and pressed into a transparent wafer. In order to verify uniformity, measurements were performed on at least three random portions of each sample. The spectra were collected within the range of 4000 cm^−1^ to 400 cm^−1^ at a resolution of 4 cm^−1^ with 32 scans [24].

#### 2.3.8. Scanning Electron Microscopy (SEM) Analysis

The SEM analysis was conducted according to the method described by Tanpichai et al. [25] with minor modifications. Briefly, raw materials and papery food dried through vacuum freeze drying were glued on the sample holder using conductive adhesive and then coated with a thin gold layer using an ion sputtering instrument (JYSC-100, Guangzhou Jingying Scientific Instrument Co., Ltd., Guangzhou, China). Microscopic images of the samples’ surface were observed under a high-resolution field emission scanning electron microscope (FEI Verios 460, FEI company, Hillsboro, USA, OSU) at a magnification of 5000× and an acceleration voltage of 5 kV [26].

### 2.4. Statistical Analysis

All experiments were run in triplicate and the results are presented as the mean and standard deviation of three replicates. Statistical analysis was performed using one-way analysis of variance (ANOVA) using SPSS software (SPSS Statistics 17.0, SPSS Inc., Chicago, USA, IL). When *p* < 0.05, differences were considered statistically significant. OriginPro 8.5 (OriginLab Corp., Northampton, MA, USA) was used for data processing and graphic construction.

## 3. Results and Discussion

### 3.1. Nutrient Composition Analysis of Pleurotus eryngii Papery Food

**Figure 1:**
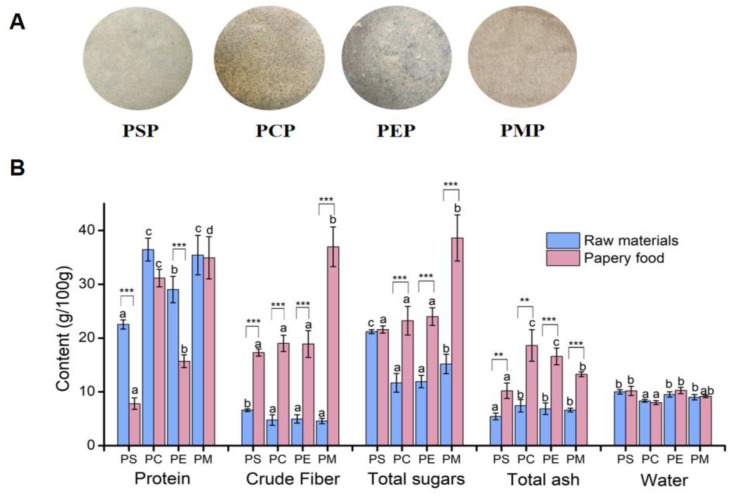
Nutritional composition of four raw materials and *P. eryngii* papery food. (**A**) Four *P. eryngii* papery foods prepared using the papermaking process. (**B**) The content of protein, crude fiber, total sugars, total ash, and water of raw materials and *P. eryngii* papery food. Each error bar indicates mean ± SD. The letters of each variable in each sample or raw material are compared. Bars sharing the same letters are not different at *p* = 0.05. Different letters indicate that the mean values of three replicates are significantly different between the treatments (*p* < 0.05). The significant differences between the raw materials and papery food were indicated by *. ** refers to significant differences where *p* < 0.05; *** refers to significant differences where *p* < 0.01.

Four *P. eryngii* papery food samples prepared using the papermaking process are displayed in Figure 1A. According to the measurement of nutritional composition, the water content, total sugar, crude protein, total ash, and crude fiber of four raw materials and four *P. eryngii* papery food are shown in Figure 1B. For the papery food, the nutritional composition changed: the protein content changed between 7.81% (PSP) and 34.93% (PMP); the total sugar content between 21.60% (PCP) and 38.61% (PSP); the crude fiber content between 17.30% (PSP) and 36.98% (PMP); the total ash content between 10.20% (PSP) and 18.63% (PCP); and the water content between 8.02% (PCP) and 10.26% (PEP). There was a significant increase in crude fiber, total sugar, and ash content in the papery food compared to the raw materials, a decrease in the protein content, and no obvious changes in the water content. Proteins and sugar were the predominant components in all of the papery foods. The crude fiber and total sugar contents of PMP were significantly higher than those of the other three groups. Furthermore, it is worth noting that PSP, PCP, and PEP only displayed distinct differences in the protein content.

Moisture content is one of the most important factors affecting crisps’ quality [27]. An excessively high water content will lead to poor crispness and storability, whereas a low content leads to high hardness and poor mouthfeel [28]. Therefore, maintaining a certain amount of moisture in the process of papery food production is very important. The results showed that the average moisture content of *P. eryngii* papery food is about 9%, which is not in line with a previous study that reported that a moisture content less than 5% maintains the crispness of crispy chips [29]. Thus, the duration of vacuum drying could be appropriately prolonged to reduce the moisture content of *P. eryngii* papery food.

The total ash was primarily reflected the content of inorganic elements such as ferrum (Fe), calcium (Ca), kalium (K), natrium (Na), magnesium (Mg), sulfur (S), silicon (Si), phosphorus (P), and aluminum (Al) [12]. The average value of total ash content in the papery food was about twice that of the raw materials (Figure 1B). The total fat content was not measured in this study as the fruiting bodies and mycelium of *P. eryngii* have an extremely low fat content and no additional cooking oil was added during the preparation of the papery food. Additionally, the lower fat content is conducive to prolonging the product shelf life, improving the taste of papery food, and is beneficial to human health [30].

The protein content of all of the papery food was reduced compared to the raw materials, which may be because some soluble proteins and polysaccharides were dissolved in water during the process of slurry disintegration, and then filtered out during the forming process. It may also be due to the Maillard reaction with polysaccharides and proteins as substrates, which was easier to occur because of a higher vacuum-drying temperature. One possible reason for the small change in the protein content of PMP is that most of the nutrients in the mycelium were insoluble proteins and polysaccharides. During the process of mycelium collection, the mycelium was washed several times with deionized water in order to completely eliminate the interference of the medium. Moreover, the reason why the total sugar content was higher in the papery foods vs. the raw materials may be due to the large increase of the crude fiber content making up for the loss of partial soluble polysaccharides. The combination of cellulose with protein and other nutrients in *P. eryngii* may be destroyed in the process of slurry disintegration and beating, resulting in an increase in the content of cellulose in all papery food [31]. Studies have shown that the daily consumption of food rich in crude fiber has beneficial effects on the human blood lipid profile, reduces low-density lipoprotein (LDL) cholesterol, and lowers triglyceride levels in women [32].

### 3.2. Crispness Analysis of Pleurotus eryngii Papery Food

**Figure 2:**
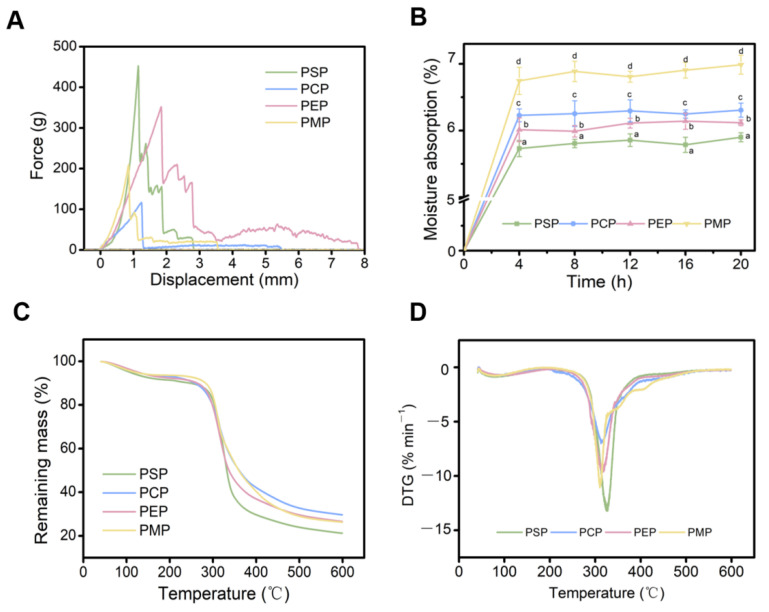
Physical properties of four *P. eryngii* papery food items. The force–displacement curves (**A**) and moisture absorption properties (**B**) of *P. eryngii* papery food. Thermogravimetric (**C**) and derivative thermogravimetric (**D**) curves of *P. eryngii* papery food. Values are expressed as mean ± standard deviation values. Different letters within the same column indicate significant differences (*p* < 0.05).

Crispness is a significant texture evaluation index of papery food [27]. Mechanical measurements using texture analyzers were the most common in previous reports [33,34]. The probe was constantly subjected to the surrounding crispy chip tissue squeeze, when the acting surface of the spherical probe increased, allowing more cracks to be generated in the crispy chip and finally more peaks to appear on the force–displacement curve. Therefore, the number of positive peaks can be used to reflect the fracture degree of the internal tissue of the papery food. In addition, a previous study suggested that the correlation coefficient between the number of spatial ruptures and the moisture content of crispy chips reached 0.96 [16], indicating that N_sr_ can be used to infer papery food crispness. The force–displacement curves of four *P. eryngii* papery foods are shown in Figure 2A. PSP had the most positive peaks with a total of eight, while PCP had the least with only two. The order of the number of positive peaks was PSP > PEP > PMP > PCP.

The changes of the papery food in the process of probe puncture can be roughly partitioned into three phases, and correspondingly, the force–displacement curve of each sample also had three phases. In the first stage, the upper surface (that is, the first time the surface made contact with the spherical probe) of the papery food was concave, while the lower surface did not change. When the internal pores of the papery food were compacted by the probe, the point with the weakest connection in the organizational structure broke first, and peaks subsequently appeared on the force–displacement curve. The second stage was an intensive phase of peak generation. On the lower surface, cracks appeared when the force on the papery food exceeded the maximum force that it could bear, which caused a sudden decrease in the force on the probe and then a large drop in the peak. The bottom of the papery food protruded outward along the cracks and the bulges became more and more obvious with the increasing puncture distance. Therefore, there were more peaks on the force–displacement curve. In the third stage, the force on the papery food presented a gradual downward trend, which was due to a gradually formed regular hole (i.e., a hole with the same diameter as the probe) in the papery food as the probe descent distance increased, so the resistance of the probe became smaller. Meanwhile, the probe sheared with the surrounding papery food to form debris, resulting in some peaks on the curve at this stage.

Since the papery food ruptures were observed in the three stages of the force–displacement curve, the number of spatial ruptures was calculated by taking the distance corresponding to the force drop to zero as the rupture distance in formula (1). It can be seen from Figure 2A that although the number of positive peaks of PEP was higher than that of PMP, the rupture distance was much larger. The number of spatial ruptures of PMP was calculated to be about 1.2 mm^−1^, while that of PEP was about 0.87 mm^−1^. Thus, the order of the number of spatial ruptures was as follows: PSP > PMP > PEP > PCP.

### 3.3. Thickness, Density, and Color Analyses of Pleurotus eryngii Papery Food

Thickness, density, and color may influence the taste quality of papery food and the purchasing desire for consumers. Table 1 shows the thickness, density, lightness (L*), redness (a*), and yellowness (b*) values of four *P. eryngii* papery food items.

The thicknesses of the papery foods are sorted from large to small, as follows: PSP, PMP, PEP, and PCP, which is consistent with the crispness results. Therefore, the thickness is directly proportional to crispness, whereas it is inversely proportional to density. In the papermaking process, the higher the mineral content of the raw material, the higher the density and lower the bulk property of the product. This statement can be corroborated by combining the papery food ash content results (Figure 1B).

The L* value is usually the first quality attribute assessed by consumers when determining product acceptance. Low L* values represent a darker color and are chiefly related to non-enzymatic browning reactions [9]. As can be seen from Table 1, the L* values were not evidently different among three papery foods from the fruiting bodies of *P. eryngii*, but were significantly higher compared to the papery food that used the mycelium as a raw material. In terms of the a* value, PMP was slightly biased toward red versus the other three groups, which was mainly associated with the ingredients for the preparation of the papery food. The b* values of the four *P. eryngii* papery foods differed modestly and skewed toward yellow.

### 3.4. Moisture Absorption Rate of Pleurotus eryngii Papery Food

Although the fruiting bodies and mycelia of *P. eryngii* have a high moisture content, most of the original water was removed in the process of preparing the papery food using the papermaking process, which generally makes the inside of the papery food form a structure with numerous small pores. Therefore, it is highly prone to absorb and regain moisture if the papery food is in a certain humidity environment during the subsequent transportation and storage, which affects its quality.

The moisture absorption rate of the four *P. eryngii* papery foods increased rapidly in the first 4 h, followed by a slow rise (Figure 2B). The hygroscopicity of the papery food in each time period was high in PMP, intermediate in PEP and PCP, and low in PSP. Therefore, the order of moisture barrier properties was PSP > PEP > PCP > PMP. In fact, although there were significant differences among the groups, the hygroscopicity of PSP, PEP, and PCP was significantly lower than that of PMP, which is probably attributed to the fact that the mycelia contain more fibers compared to the fruiting bodies of *P. eryngii*, and the linear fibers are intertwined to form a complex network with numerous pores. The difference in the hygroscopicity of the four *P. eryngii* papery foods is closely related to its internal structure with small pores. In general, the larger the specific surface area of the pores, the more sensitive the papery food is to the humidity in the environment [35]. One study linked the hygroscopic isotherm of a freeze-dried orange snack to its mechanical properties. It found that storage at atmospheres with a relative humidity higher than 32% would lead to the loss of its crispness [36]. One cause for this result was that a significant positive correlation (r = 0.67, *p* < 0.001) was obtained between porosity and the number of force peaks [16]. In this sense, *P. eryngii* papery food should be stored in an environment where the relative humidity (RH) is less than 30% to effectively avoid the decrease in quality caused by the reduction of porosity and the loss of crispness.

### 3.5. Thermal Degradation Properties of Pleurotus eryngii Papery Food

The thermal degradation properties of papery food were studied by thermogravimetric analysis. Figure 2C,D present thermogravimetric (TG) and derivative thermogravimetric (DTG) curves of the four *P. eryngii* papery food items. Displaying typical thermal degradation and combustion reaction characteristics of organic materials, the results indicate that the thermogravimetric analysis curves of the four papery foods can be roughly divided into three stages [37]. Initially, all samples lost approximately 10% of their weights between 40 and 250 °C, probably due to the evaporation of free and chemically bonded water. A greater mass loss (about 50 wt%) then occurred between 280 and 380 °C, which was ascribed to the degradation of the organic constituents such as polysaccharides and proteins [22]. The final mass loss from 400 to 600 °C was associated with further thermal degradation of the primary char residue, which produced many final carbonaceous char residues [38]. The existence of hemicellulose and lignin affects the thermal degradation performance of papery food. A higher amount of char residue at a high temperature was found from lignin than from cellulose and hemicellulose [39]. It is worth mentioning that some differences in the residue content between PSP and PCP was observed. The PCP yielded the highest char residues (around 30 wt%), suggesting the significant biomineralization and the lower organic content of PCP compared to that of the other three samples. Additionally, the PSP exhibited only a final inorganic residue of approximately 20 wt%, which was likely owing to the higher cellulose content. Herein, the TG results appeared in accordance with those obtained from the nutrient components.

Derivative thermogravimetric analysis curves indicated that the DTG peak temperature did not vary greatly among the four *P. eryngii* papery foods. In detail, the PMP showed the lowest DTG peak temperature at 309.9 °C, demonstrating an inferior thermal stability compared to the papery food prepared from the fruiting bodies of *P. eryngii*. The DTG peak temperature of the PSP was found to be slightly higher than that of PEP and PCP due to a higher lignin content in the PSP, suggesting that PSP has the strongest thermal stability.

### 3.6. Sensory Evaluation of Pleurotus eryngii Papery Food

**Figure 3:**
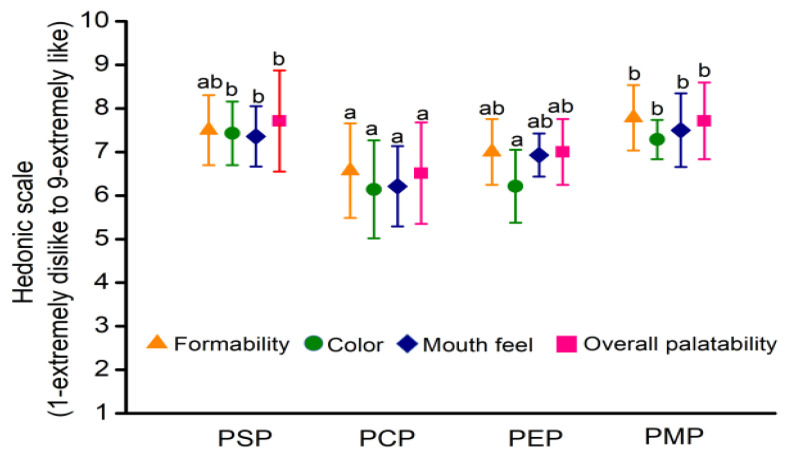
Sensory attributes of four *P. eryngii* papery food items. Each error bar indicates mean ± SD. Bars sharing the same letters are not different at *p* = 0.05. Letters should be compared for each variable across treatments. Different letters indicate that the mean values of three replicates are significantly different between the treatments (*p* < 0.05).

In the food industry, sensory evaluation of the product gives a scientific assessment and a quantitative measure to the appearance, flavor, and texture of the product, which is vital to studying the overall preferences of the consumer [40]. Multiple sensory attributes are linked to palatability. Sensory acceptance was assessed in this study for formability, color, mouth feel, and overall acceptability of four *P. eryngii* papery foods (Figure 3).

All parameters of PCP were lower than those of the other three groups. PMP scored better in formability (7.79), which may be because the mycelium was more easily dispersed into fibrous material than the fruiting bodies in the beating process, resulting in its uniform distribution during the precipitation process. A primary reason for the lower consumer preference for PCP and PEP is the uneven color of the *P. eryngii* cap, caused by the fact that the surface color of *P. eryngii* caps was different from the inside color and puncta was formed when exposed to sunlight during growth. The decrease in the mouth feel of PCP might be responsible for the poor thickness and crispness that were caused by the looser and weaker tissue of *P. eryngii* caps. There was no significant difference in the overall palatability between PSP and PMP, which was slightly better than that of PEP and PCP. The average scores of the sensory acceptance of the four *P. eryngii* papery foods showed that PMP (7.43) and PSP (7.52) were more popular among consumers. PMP and PSP exhibited either good or excellent color and morphology, a crisp texture, and the characteristic flavor and taste of *P. eryngii*. In addition, the factors affecting the papery food quality may include blender rotations, vacuum-drying temperature, and drying time, and sensory aspects may be improved by the adjustment of the above processes’ parameters.

### 3.7. FT-IR Analysis of Pleurotus eryngii Papery Food

**Figure 4:**
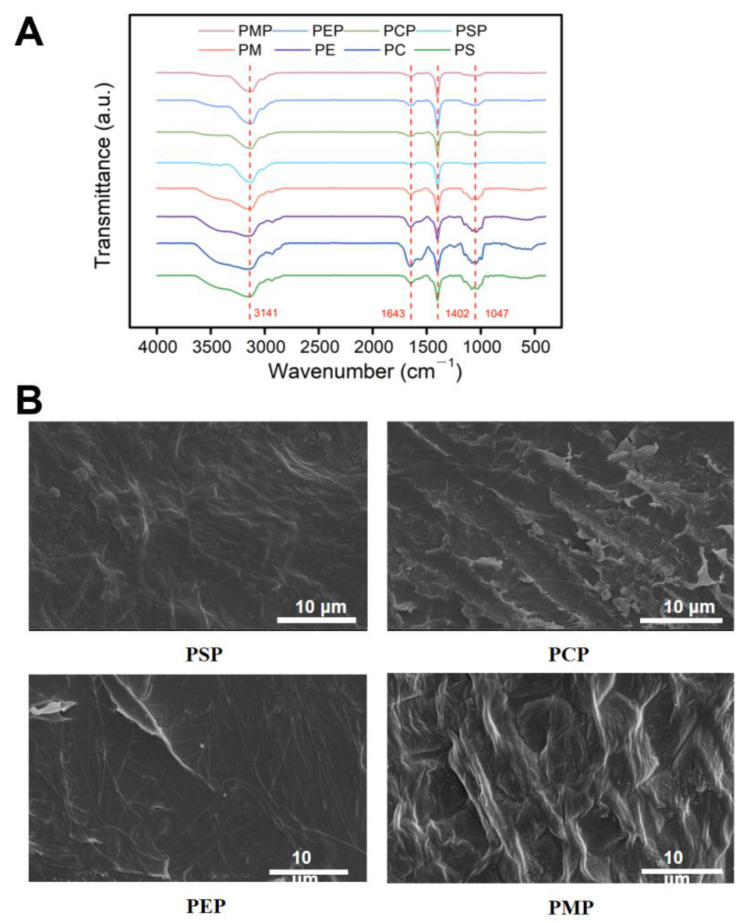
Chemical structure and micromorphology of four *P. eryngii* papery food items. (**A**) The FT-IR spectra of raw materials and *P. eryngii* papery foods. (**B**) SEM images of the surfaces of four *P. eryngii* papery foods (PSP, PCP, PEP, and PMP). The magnification level was 5000×.

The chemical structure of the materials was analyzed by FT-IR spectroscopy. The FT-IR spectra of the papery food are shown in Figure 4A along with those of the raw materials. The fruiting bodies and mycelium of *P. eryngii* are mainly composed of proteins and saccharides, followed by minerals, vitamins, and bioactive compounds such as triterpenoid. In addition, the self-grown mycelium was reported to contain nucleic acids including phosphate, sugar, and nitrogenous bases [41,42]. The *P. eryngii* papery food, on the other hand, consisted of predominantly dietary fiber (cellulose, lignin, hemicellulose, and galactan) and proteins.

All raw materials and papery foods presented similar FT-IR spectra, suggesting the similar main composition in the samples, which were proteins and polysaccharides such as cellulose, hemicellulose, and lignin. No additional characteristic absorption peaks were generated for any of the papery foods after the papermaking process, which indicates that no new functional groups were formed in the preparation of the papery food. In the graph, all samples presented an amide I band associated with C=O stretching around 1643 cm^−1^ and amide II and III bands resulting from −NH deformation near 1402 cm^−1^. The typical signal of −OH stretching of polysaccharides (from cellulose and hemicellulose) at 3141 cm^−1^ could be observed. The absorption bands around 1047 cm^−1^ are related to the stretching vibrations of the −C−O−C− and −OH groups from polysaccharide. Signals around 1240 cm^−1^ and 1550 cm^−1^ related to the aromatic skeletal vibration of lignin were clearly visible in papery food.

### 3.8. Microstructure of Pleurotus eryngii Papery Food

The effects of *P. eryngii* mycelium and different parts of the fruiting bodies on the preparation of papery food were investigated using SEM. The surface morphology of the four *P. eryngii* papery foods are shown in Figure 4B. The surface of PCP exhibited rough morphology and uneven thickness with many small solid particle protrusions. Conversely, the surface of PSP was relatively flat and homogeneous, and presented few protrusions in local areas, suggesting that the *P. eryngii* stems were evenly dispersed before papermaking. In the PEP and PMP samples, dispersed fine fibers were randomly deposited without any preferred orientation, resulting in large pores among the fibers. The above observations by SEM are in agreement with the results of formability in sensory evaluation. The addition of food thickeners might fill the cavities within papery food, thereby making the surface appear to be smoother. Moreover, some studies have shown that with the addition of sodium alginate, carboxymethylcellulose sodium (CMC-Na), and other hydrocolloids, the surface of papery food become flatter and more uniform with a brighter color, with increased tensile strength [43]. Nevertheless, as the pore size of the filter screen of the paper sheet former is small whether the addition of food thickeners has an influence on the papermaking process still needs to be further investigated.

## 4. Conclusions

Papery food is widely consumed due to its palatability, portability, and long shelf life. In this study, the papermaking technology was combined with food processing to prepare *P. eryngii* papery food with a flat appearance and uniform moisture distribution, which preserved its mycelium and fruiting bodies nutrition and flavor to the maximum extent.

The present work aimed to conduct an in-depth assessment of the physical, chemical, and structural properties and sensory attributes. The results suggested that all of the papery foods were rich in polysaccharides (22.39% on average) and proteins (22.39% on average) and the color of the papery foods made from the fruiting bodies of *P. eryngii* was obviously different from that made from the mycelium. In general, PSP had a flatter surface and better crispness, moisture resistance, and thermal degradation. Additionally, the average score of the sensory attributes of PMP and PSP reached 7.43 and 7.52, respectively (full score of 9), which were the most preferred among the consumers. Furthermore, it is worth mentioning that this study may provide ideas for the construction of an edible fungi papery food factory. The present study put forward a new idea for the processing of edible fungi products, which provides a theoretical reference for future edible fungi food factories. However, this study only studied *P. eryngii* papery food, and the influence of additives including food gums and edible filter aids on the papermaking process and finished products would be the focus for future study.

## Figures and Tables

**Table 1:** Thickness, density, and chromatic values of four *P. eryngii* papery foods.

Samples	Thickness (mm)	Density (g cm^−3^)	Chromatic Values		
			L*	a*	b*
PSP	0.32 ± 0.022 ^d^	0.38 ± 0.014 ^a^	75.54 ± 0.351 ^b^	1.66 ± 0.048 ^a^	20.71 ± 0.281 ^a^
PCP	0.15 ± 0.016 ^a^	0.81 ± 0.073 ^c^	77.45 ± 0.196 ^b,c^	3.42 ± 0.064 ^c^	25.58 ± 1.505 ^b^
PEP	0.19 ± 0.012 ^b^	0.64 ± 0.013 ^b^	80.93 ± 2.276 ^c^	2.26 ± 0.177 ^b^	21.61 ± 1.094 ^a^
PMP	0.26 ± 0.008 ^c^	0.47 ± 0.010 ^a^	17.02 ± 1.653 ^a^	6.28 ± 0.067 ^d^	28.62 ± 0.492 ^c^

Note: Values are expressed as mean ± standard deviation. Different letters within the same column indicate significant differences (*p* < 0.05).

## Data Availability

Data is contained within the article.
